# Prevalence of Antibiotic-Resistant *Escherichia coli* Isolated from Beef Cattle and Dairy Cows in a Livestock Farm in Yamagata, Japan

**DOI:** 10.3390/microorganisms12071342

**Published:** 2024-06-30

**Authors:** Tumurbaatar Khishigtuya, Hiroki Matsuyama, Kazuhito Suzuki, Toru Watanabe, Masateru Nishiyama

**Affiliations:** 1 The United Graduate School of Agricultural Sciences, Iwate University, 18-8 Ueda 3-chome, Morioka 020-8550, Iwate, Japan; khishigtuyat@gmail.com; 2 Faculty of Agriculture, Yamagata University, 1-23 Wakaba-machi, Tsuruoka 997-8555, Yamagata, Japan; matsuyama@tds1.tr.yamagata-u.ac.jp (H.M.); to-ru@tds1.tr.yamagata-u.ac.jp (T.W.); 3 Yamagata Prefecture Livestock Research Institute, 1076 Ipponmatsu, Torigoe, Shinjo 996-0041, Yamagata, Japan; suzukikazuh@pref.yamagata.jp

**Keywords:** *Escherichia coli*, antibiotic-resistant, feces, beef cattle, dairy cow

## Abstract

Antimicrobials are used on livestock farms to treat and prevent infectious animal diseases and to promote the growth of livestock. We monitored the prevalence of antibiotic-resistant *Escherichia coli* (AR-EC) isolates from beef cattle (BC) and dairy cows (DCs) on a livestock farm in Yamagata, Japan. Fecal samples from 5 male BC and 10 male DCs were collected monthly from October 2022 to November 2023. In total, 152 and 884 *E. coli* isolates were obtained from the BC and DC fecal samples, respectively. Notably, 26 (17.1%) and 29 (3.3%) *E. coli* isolates in the BC and DC groups, respectively, were resistant to at least one antibiotic. The resistance rates to tetracycline, ampicillin, gentamicin, and chloramphenicol of the isolates were significantly higher than those to the other antimicrobials. The tetracycline resistance genes *tetA* (70.6%) in DCs and *tetB* (28%) in BC were identified, along with the *bla*_TEM_ gene in ampicillin-resistant isolates (BC: 84.2%, DCs: 42.8%). Despite significant variations in the monthly detection rates of AR-EC isolated from BC and DCs throughout the sampling period, the judicious use of antimicrobials reduced the occurrence of AR-EC in both BC and DCs, thereby minimizing their release into the environment.

## 1. Introduction

Since the last century, the use of antibiotics has significantly increased life expectancy. However, the overuse and misuse of these antimicrobials have led to the development of antibiotic-resistant bacteria (ARB). ARB are a growing concern for global public health and are recognized by the World Health Organization [1] as one of 21st century’s biggest threats. According to estimates, the annual death toll from ARB was 700,000 worldwide in 2014 and is projected to exceed 10 million by 2050 [2]. By 2050, the mortality rate due to ARB per 10,000 people in Africa and Asia is expected to be approximately twice as high as that in North America, Europe, and Australia [3]. Conditions such as tuberculosis and gonorrhea and bacteria such as *Escherichia coli* are becoming increasingly resistant to treatment [3]. 

Inappropriate antibiotic use, both in excessive and in limited quantities, in humans and livestock causes ARB. Globally, more than 60,000 tons of antibiotics are consumed annually by large-scale livestock operations, leading to the evolution of ARB that can be transmitted to humans [3]. Antibiotic use in livestock farming is higher than in humans, making livestock a hotspot for ARB emergence because of the frequent use of antimicrobial agents. The top five antimicrobial consumers in 2020 were China, Brazil, India, the United States of America, and Australia [4]. In Japan, the amount of antibiotics used for livestock, including feed additives, totaled 834.1 tons per year, exceeding the 626.8 tons per year used by humans [5]. Veterinary antimicrobials and feed additives constitute over half of the antimicrobials consumed in livestock production in Japan [5]. Therefore, future studies should consider the use of antibiotics as feed additives for livestock. 

Livestock disease management and growth promotion are essential activities on livestock farms; however, they also contribute to the occurrence of ARB. Livestock farms are recognized as significant sources of ARB, which can be transmitted to humans via meat consumption [6]. The appropriate administration and management of antibacterial substances can help suppress the spread of ARB. Using antibiotics only when necessary and at the correct dose can significantly reduce the emergence and spread of ARB [7]. 

Moreover, ARB can be excreted and discharged into the environment, posing the hazard of AR spreading. Therefore, antibiotics must be used responsibly, and measures must be taken to prevent their spread into the environment [8]. The WHO and the Centers for Disease Control and Prevention (CDC) have published research highlighting the severity of ARB [9]. Antibiotics are critical for treating bacterial infections in humans and animals [10]. However, research on differences in AR prevalence between beef cattle (BC) and dairy cows (DCs) on a single farm throughout the year is lacking.

Therefore, this study aimed to analyze variations in antibiotic-resistant *E. coli* (AR-EC) in BC and DCs, specifically in the same individuals, continuously throughout the year, in response to different antimicrobial agents.

## 2. Materials and Methods

### 2.1. Sampling

**Figure A1:**
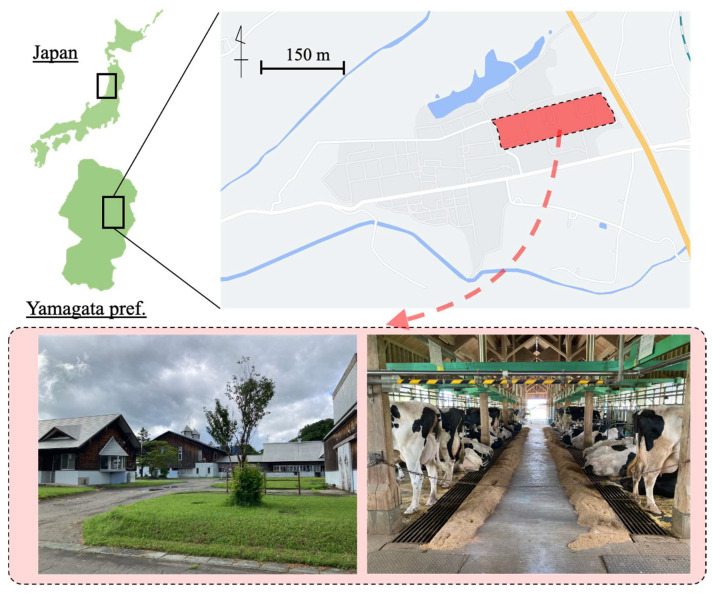
Location of the farm.

Sampling was conducted at the Yamagata Prefecture Livestock Research Institute, located in Shinjo city in a mountain basin in the northeast of Yamagata prefecture in the southwest corner of Tōhoku region, Japan (Appendix A Figure A1). The barns of BC and DCs were separated on the farm, and the number of BC and DCs reared during the study period averaged 10 and 20 heads monthly. Fecal samples were collected monthly from five male BC (aged 8–9 months) and 10 DCs (aged 29–60 months) between October 2022 and November 2023 at the Research Center on Livestock, Yamagata Prefecture Government, Japan. At this farm, DCs are fed grass silage, corn silage, compound feed manufactured in Japan, and imported feed, such as dried grass, hay cubes, and beet pulp. They also receive vitamins and mineral compounds in a tie-stall barn with separate feeding. In contrast, BC are fed rice straw from Japan, compound feed manufactured in Japan, and corn silage. DCs receive feed-grade glycerine and bypass fat supplements, whereas BC do not. During the study period, nine DCs showed symptoms of mastitis, and some antimicrobials were injected for treatment. Information regarding the administration of the antimicrobials is presented in Table 1. None of the BC were treated with antimicrobials. Wild birds and raccoon dogs have been observed on farms; however, no unauthorized entry into cattle barns has been reported. However, there is a possibility of mice entering the barns. No new cattle were introduced to the farm. 

Fecal samples (>10–50 g) were collected directly from the BC and DCs by rectal palpation and transported in a cooler box (4–8 °C) to the laboratory on the same day.

### 2.2. Bacterial Counting, Isolation, and Identification of E.coli

The number of *E. coli* in the fecal samples from both BC and DCs was determined using the plate dilution method. Specifically, 1 g of each fecal sample was diluted with 9 mL of sterile saline to achieve a 10^−5^ dilution. Subsequently, 0.1 mL of a 10^−1^–10^−5^ dilution was spread on an agar plate (Chromocult Coliform; Sigma Aldrich, Germany). For *E. coli* screening, the diluted samples were smeared on CHROMagar^TM^ extended-spectrum β-lactamase (ESBL)-producing Enterobacterales, vancomycin-resistant Enterococcus (VRE), and carbapenem-resistant Enterobacterales (CRE) agar plates (Kanto Chemical, Tokyo, Japan). The plates were incubated at 37 °C for 24 h, and the positive colonies on the agar were counted. Colony counts were calculated as the average number of colony-forming units (CFUs) in triplicate. Up to 10 of the presumptively identified blue colonies of *E. coli* were picked from the Chromocult Coliform and streaked on Luria–Bertani agar (LB, Difco^TM^ LB Broth, Lennox, USA, agar powder, Fujifilm Wako Pure Chemicals Co., Ltd., Osaka, Japan). After incubation at 37 °C for 24 h, the colonies grown on LB agar were purified by streaking onto the same agar. Purified colonies were isolated in LB broth and stored at −80 °C until further analysis.

DNA of the suspected positive *E. coli* isolates was extracted using the InstaGene Matrix (Bio-Rad Laboratories, Inc., Hercules, CA, USA) according to the manufacturer’s protocol. Presumptive E. coli isolates were identified by detecting a specific gene for *E. coli* (*uidA*) using a KAPATaq EXtra PCR kit (NIPPON Genetics Co., Ltd., Tokyo, Japan) [11]. The PCR conditions were as follows: three minutes at 95 °C for initial denaturation, 35 cycles of 30 s each at 95 °C for denaturation, 30 s at 58 °C, and one minute at 72 °C for elongation, followed by 10 min at 72 °C for final elongation using a Bio-Rad T100TM Cycler (USA) (Appendix A Table A1). The PCR products (2.5 μL) were mixed with 0.5 μL of 6 × GR Green Loading Buffer (Biocraft, Tokyo, Japan) and confirmed by 1.5% agarose gel electrophoresis using Tris–borate–EDTA buffer (Takara, Shiga, Japan). *E. coli* NBRC3301 was used as a positive control for PCR analysis.

### 2.3. Antibiotic Susceptibility Test 

Single-well isolated colonies from Luria–Bertani agar were emulsified in 10 mL of sterile saline and adjusted to a McFarland standard 0.5 (bioMérieux SA Ltd., Marcy l’Etoile, France). A sterile cotton swab was dipped in a standardized bacterial suspension and evenly streaked over the entire surface of Mueller–Hinton agar. Impregnated paper discs with a constant concentration of antibiotics were placed on the surface of the agar and incubated at 37 °C for 16–18 h. Subsequently, *E. coli* isolates were tested for susceptibility to 18 antibiotics (cefotaxime [30 µg], ceftazidime [CAZ, 30 µg], cefpodoxime [10 µg], ampicillin [ABP, 10 µg], cefuroxime [CXM, 30 µg], cefoxitin [10 µg]; imipenem [10 µg], aztreonam [30 µg], amoxicillin and clavulanic acid [20:10 µg), tazobactam/piperacillin (T/P, 100 µg/10 µg], sulfamethoxazole/trimethoprim [ST, 23.75 µg/1.25 µg], gentamicin [GM, 10 µg], amikacin [AMK, 30 µg], ciprofloxacin [5 µg], tetracycline [TC, 30 µg], tigecycline [15 µg], fosfomycin [200 µg], and chloramphenicol [CP, 30 µg]) that were selected based on the Clinical and Laboratory Standards Institute (CLSI) guidelines, using the disc diffusion method of Kirby–Bauer, and their resistances were determined following the standards of the CLSI M100-ED33:2023 [12]. *E. coli* ATCC 25,922 was included in all antimicrobial susceptibility tests (ASTs) for quality control.

Resistance to ≥3 antibiotic classes was used to classify the bacterial isolates as multidrug-resistant (MDR).

### 2.4. Detection of Antibiotic Resistance Genes

To characterize the TC- and ABP-resistant *E. coli* isolates, the *tet* and *bla* genes corresponding to the TC and ABP resistance genes were identified using PCR. DNA was extracted from the isolates. The primers used for each targeted antibiotic resistance gene and the PCR amplification program are described in Appendix A Table A1 [13,14,15]. The enzyme KAPATaq Extra was used for PCR amplification of the ARGs. PCR amplification was confirmed by electrophoresis as described above. 

### 2.5. Statistical Analysis

To examine statistical differences in the proportions of AR-EC isolates, we used the Z-test and the correlation coefficient in Excel. Additionally, one-way analysis of variance (ANOVA) and Tukey’s honestly significant difference analyses were performed using SPSS (version 22) statistical software.

## 3. Results and Discussion 

### 3.1. E. coli Concentrations in Feces

**Figure 1:**
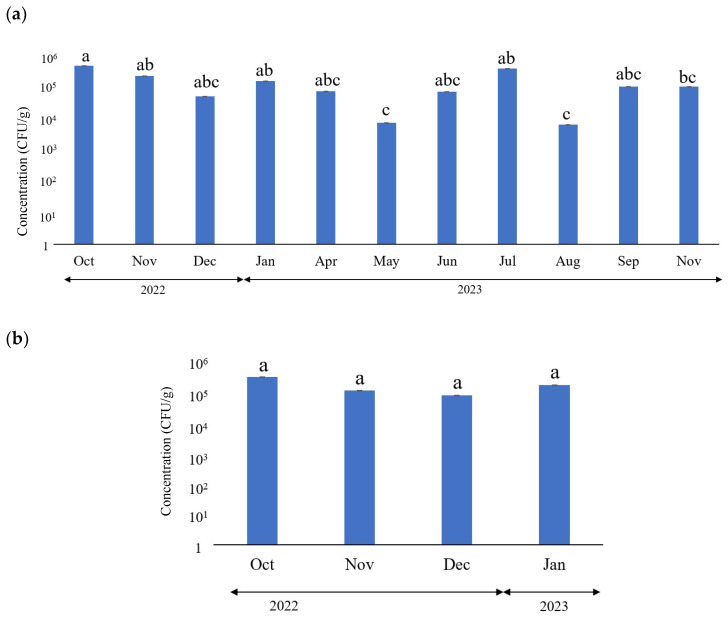
Monthly *Escherichia coli* concentrations in the feces of (**a**) dairy cows (DCs) and (**b**) beef cattle (BC).

Figure 1 shows the monthly *E. coli* concentrations in the fecal samples obtained from BC and DCs. According to the WHO, carbapenem- and β-lactam-resistant bacteria and vancomycin-resistant enterococci are classified as serious ARB. None of these three resistant strains (ESBL, CRE, and VRE) were detected on any of the media for AR bacteria, indicating that the farm effectively managed antibiotic usage.

No significant differences were observed in *E. coli* concentration between BC (3.33 × 10^3^–2.7 × 10^6^ CFU/g) and DCs (1.7 × 10^2^–7.9 × 10^6^ CFU/g). Furthermore, the *E. coli* concentrations in BC and DCs did not depend on seasonal changes. However, the *E. coli* concentrations were significantly higher in October and November 2022 and January and July 2023 than in May and August 2023 (Figure 1). This may be associated with lower concentrations of antimicrobial medications or lower quality sanitation facilities in May and August. 

### 3.2. Antibiotic Susceptibility Test

A total of 152 and 884 *E. coli* isolates, identified by the presence of the *uidA* gene using PCR, were obtained from fecal samples of BC and DCs, respectively. All isolates harbored the *uidA* gene and were subsequently identified as *E. coli*. 

All isolates underwent ASTs using 18 antimicrobial agents. Notably, 26 (17.1%) and 29 (3.3%) *E. coli* isolates from BC and DCs, respectively, exhibited resistance to at least one antibiotic. The highest resistance rates were observed for TC (BC, 16.5%; DCs, 11.2%), ABP (BC, 12.5%; DCs, 4.6%), GM (BC, 11.8%; DCs, 0%), and CP (BC, 9.9%; DCs, 0%), which are commonly used for disease treatment. The resistance rates of the isolates to other antimicrobials were generally low, as follows: AMK (BC, 0%; DCs, 3.3%), ST (BC, 0%; DCs, 2.6%), CIP (BC, 0%; DCs, 2.6%), T/P (BC, 0%; DCs, 1.3%), CXM (BC, 0%; DCs, 0.7%), and CAZ (BC, 0.7%; DCs, 0%). According to reports from other countries, the most frequently observed antimicrobial resistance in *E. coli* isolates is to tetracycline [16,17]. The resistance rates to TC were 6% lower than those reports, and that to ABP was similar to that reported in the Nippon Antimicrobial Resistance (AMR) One Health Report 2022 [5]. In 2018, Japan’s national drug resistance statistics reported a 26.5% tetracycline resistance rate in healthy cattle on livestock farms [10]. However, studies conducted in Asia, the U.K., and the U.S.A. have revealed much higher resistance rates in dairy cows, ranging from 33.3% to 93% [18,19,20]. These variations in resistance rates can be attributed to factors such as infectious diseases incidents, treatment protocols, and geography. The high rates of tetracycline resistance are linked to its extensive use in both human medicine and animal husbandry. Tetracycline is favored for its affordability and minimal side effects. The correlation coefficient was employed to assess the correlation between time and AR rate. However, no significant correlation was observed between time and AR rates in either animal type. The monthly detection rates of AR-EC isolated from the BC and DCs varied considerably throughout the sampling period (Table 2).

The antibiotic resistance rates of the *E. coli* isolates were not correlated with head or injection history (Table 1). The antimicrobials and injections used for the DCs were different, except for CXM. The prevalence of AR-EC can be attributed to many factors such as drug administration, feed additives, farm management, farm hygiene, and farm size. The emergence of AR involves the administration of antibiotics and other poorly understood factors [21]. One reason for the prevalence of AR-EC is that farm hygiene problems such as forage and haylage can lead to fecal matter contamination from wild animals [22]. Farm size is crucial for the prevalence of AR-EC. Increasing the number of cattle increases the incidence of AR-EC. Furthermore, farms that buy more cattle have a higher likelihood of detecting ESBL-producing *E. coli* [23]. The prevalence of antimicrobial resistance among isolates collected from calves is thought to be greater than that among those collected from cows, as calves receive antimicrobial treatment more frequently than lactating dairy cows [24].

### 3.3. Profiling of AR-EC Multidrug Resistance

Based on their AR patterns, 152 and 884 *E. coli* isolates from BC and DCs belonged to five and seven different phenotypes, respectively. These ranged from resistance to a single antimicrobial to a combination of four phenotypes (Table 3). The most frequently observed phenotype was ABP-GM-TC, with a prevalence of 11.8% (18 isolates), followed by ABP-GM-TC-CP (9.2%; 14 isolates), TC (3.9%; 6 isolates), ABP-TC-CP (1.3%; 2 isolates), and CAZ (0.7%; 1 isolate) in BC. In 2020, antimicrobial resistance rates exceeding 50% were observed for ABP, streptomycin, and TC in deceased cattle [5]. The most frequently observed phenotype in DCs was TC, with a prevalence of 1.5%, followed by ABP-ST-TC, CIP, and AMK at 0.5% each, and ABP-T/P-AMK, ABP, and T/P at 0.1% each. In general, multidrug AR-EC in BC was higher than that in DCs. This may be because antibiotics are mainly used to treat mastitis on dairy farms; however, ARB in mastitis-causing pathogens are relatively infrequent [25]. The TC resistance rates were almost four times lower than those in the U.S.A. (25%) and five or more times lower than those in other countries (Canada, 31%; Germany 36%; France 52%; Italy 79%), according to data from the National Action Plan on AMR (2023–2027) [26]. Furthermore, MDR strains with resistance to as many as four antibiotics in this study were fewer than those reported in a Japanese report [27], with more than six antibiotics, and in other countries. The U.S.A. reported MDR to more than six antibiotics [28], Canada to five [29], and Korea to six [30]. MDR strains are more frequently found in calves than in growing or mature cattle in various research studies [24,31]. These findings suggest that calves are an optimal source of ARB. This notion is further supported by the negative correlation between the prevalence of resistant *E. coli* and the age of the source animal [32]. Furthermore, Gow et al. indicated that ARB in calves could be transmitted among cattle, given the similarity in AMR patterns between cows and calves [31]. The low prevalence of MDR strains in the studied farm may be associated with well-managed drug administration.

### 3.4. Detection of Antibiotic-Resistant Genes

Based on the AST results, 42 isolates were resistant to TC (BC: 16.5%, DCs: 11.2%), and 26 isolates were resistant to ABP (BC: 12.5%, DCs: 4.6%). These numbers were higher than those for the other tested antibiotics. Nine TC resistance-related genes and five ABP resistance-related genes were identified.

Notably, *tet*C, *tet*D, *tet*E, *tet*G, *tet*J, *tet*M, and *tet*W were not detected in any of the monthly samples from BC or DCs. However, *tet*A and tetB, associated with efflux pumps, were detected in 11.7% and 3.4% of the isolates, respectively. Furthermore, *tet*A (BC: 0% [0/25], DCs: 70.6% [12/17]) was detected in all TC-resistant strains, with a significant difference between the two groups (Table 4). The *tet*B gene (BC: 28% [7/25], DCs: 0% [0/17]) was detected in all TC-resistant strains, and the result was not statistically different between the two types of animals. The prevalence rates of *tet*A and *tet*B reported by Shin et al. were 24.1% lower and 17.1% higher, respectively, in BC compared to those in this study [33]. Furthermore, *tet*A and *tet*B were detected in BC in Japanese studies [27]. The high prevalence of TC resistance in *E. coli* is probably due to the horizontal transfer of *tet* determinants from *E. coli* isolates carrying *tet* genes that survive the selective pressure exerted by TC derivatives [33]. Therefore, understanding the origin and transmission route of *E. coli* in cattle is crucial to mitigate its prevalence.

Notably, the *bla*_CTX-M-1_, *bla*_CTX-M-2_, *bla*_CTX-M-8_, and *bla*_CTX-M-9_ genes were not detected in the monthly samples (Table 5). However, the antibiotic-resistant gene *bla*_TEM_ was detected in 16.3% of the livestock. Furthermore, *bla*_TEM_ (BC: 84.2% [16/19], DCs: 42.8% [3/7]) was detected in all ABP-resistant strains, and the result was not significantly different between the two groups. The extended-spectrum β-lactamase (ESBL) genes *bla*_TEM_ and *bla*_SHV_ were initially identified in the 1980s and were the most prevalent genes until 2000 [33]. Currently, ESBL production, particularly associated with *bla*_TEM_, is considered one of the most significant ARB mechanisms from both clinical and epidemiological perspectives [33]. Previous studies indicated that *bla*_TEM_ was detected in 78.9% of the isolates from dairy cattle farms in the Nile Delta, Egypt, whereas *bla*_SHV_ and *bla*_OXA_ were only detected in 0.87% of the isolates [34]. Notably, *bla*_TEM_ was detected in dairy cows [35] and remains the most common ARG in China and other countries, regardless of whether the isolates are from dairy cows or BC [36].

## 4. Conclusions

Our study indicated that the prevalence of AR-EC isolated from BC and DCs at the Yamagata Prefecture Livestock Research Institute was low or similar to that reported in Japan and other countries. This is probably because the surveyed farms were well managed by the prefectural government. Generally, the antimicrobial resistance rates in BC are higher than those in DCs. This difference was attributed to the higher prevalence of antimicrobial resistance among isolates obtained from calves, as calves tend to receive antimicrobial treatments more frequently than lactating dairy cows. The prudent use of antimicrobials contributes to reducing the occurrence of AR-EC in BC and DCs and, consequently, their release into the environment. This study provides fundamental information about ARB and contributes to improving food safety and to promoting the careful use of antimicrobial agents.

## Figures and Tables

**Table 1:** Antibiotic resistance rates (%) of *E. coli* isolates in individual BC and DC heads.

Name	CTX	CAZ	CPX	ABP	CXM	CFX	IPM	AZT	ACV	T/P	ST	GM	AMK	CIP	TC	TGC	FOM	CP	Injection History
B1 (n = 40)	0	0	0	15.0	0	0	0	0	0	0	0	15.0	0	0	25.0	0	0	7.5	-
B2 (n = 21)	0	0	0	0	0	0	0	0	0	0	0	0	0	0	0	0	0	0	-
B3 (n = 40)	0	0	0	15.0	0	0	0	0	0	0	0	12.5	0	0	20.0	0	0	12.5	-
B4 (n = 36)	0	0	0	0	0	0	0	0	0	0	0	0	0	0	0	0	0	0	-
B5 (n = 15)	6.7	0	0	46.7	0	0	0	0	0	0	0	46.7	0	0	46.7	0	0	46.7	-
D1 (n = 102)	0	0	0	0	0	0	0	0	0	0	0	0	0	0	11.8	0	0	0	C
D2 (n = 94)	0	0	0	0	0	0	0	0	0	0	0	0	0	0	0	0	0	0	SE, P
D3 (n = 87)	0	0	0	1.1	0	0	0	0	0	0	0	0	0	0	0	0	0	0	SE, PK, P, CXM, C
D4 (n = 101)	0	0	0	0	0	0	0	0	0	0	0	0	1.0	0	0	0	0	0	SE, P, CXM
D5 (n = 60)	0	0	0	0	0	0	0	0	0	0	0	0	0	0	0	0	0	0	P, C, PK
D6 (n = 86)	0	0	0	0	0	0	0	0	0	0	0	0	0	2.3	0	0	0	0	SE, SL, P
D7 (n = 110)	0	0	0	0	0	0	0	0	0	0	0	0	0	0	0	0	0	0	SE, P
D8 (n = 78)	0	0	0	6.4	0	0	0	0	0	1.3	5.1	5.1	0	0	6.4	0	0	0	-
D9 (n = 106)	0	0	0	0	0	0	0	0	0	0	0	0	0	1.9	0	0	0	0	SE, PK, C
D10 (n = 60)	0	0	0	0	0	0	0	0	0	1.7	0	0	0	0	0	0	0	0	SE, C
DCs (n = 884)	0	0	0	4.6	0.7	0	0	0	0	1.3	2.6	0	3.3	2.6	11.2	0	0	0	
BC (n = 152)	0	0.7	0	12.5	0.0	0	0	0	0	0	0	11.8	0	0	16.5	0	0	9.9	

Abbreviations: CTX, cefotaxime; CAZ, ceftazidime; CPX, cefpodoxime; ABP, ampicillin; CXM, cefuroxime; CFX, cefoxitin; IPM, imipenem; AZT, aztreonam; ACV, amoxicillin and clavulanic acid; T/P, tazobactam/piperacillin; ST, sulfamethoxazole/trimethoprim; GM, gentamicin; AMK, amikacin; CIP, ciprofloxacin; TC, tetracycline; TGC, tigecycline; FOM, fosfomycin; CP, chloramphenicol; SE: cefazolin; PK: kanamycin; P: pirlimycin hydrochloride hydrate; C: cephalonia.

**Table 2:** Antibiotic-resistance rates (%) of *E. coli* isolates to the tested antimicrobials.

Name	BC				DCs										
	Oct-22	Nov-22	Dec-22	Jan-23	Oct-22	Nov-22	Dec-22	Jan-23	Apr-23	May-23	Jun-23	Jul-23	Aug-23	Sep-23	Nov-23
	n = 36	n = 46	n = 40	n = 30	n = 100	n = 100	n = 90	n = 97	n = 81	n = 52	n = 80	n = 80	n = 64	n = 74	n = 66
CTX	0	0	0	0	0	0	0	0	0	0	0	0	0	0	0
CAZ	0	2.2	0	0	0	0	0	0	0	0	0	0	0	0	0
CPX	0	0	0	0	0	0	0	0	0	0	0	0	0	0	0
ABP	22.2	23.9	0	0	1	1	1.1	0	0	0	0	0	0	0	6.1
CXM	0	0	0	0	0	1	0	0	0	0	0	0	0	0	0
CFX	0	0	0	0	0	0	0	0	0	0	0	0	0	0	0
IPM	0	0	0	0	0	0	0	0	0	0	0	0	0	0	0
AZT	0	0	0	0	0	0	0	0	0	0	0	0	0	0	0
ACV	0	0	0	0	0	0	0	0	0	0	0	0	0	0	0
T/P	0	0	0	0	0	0	1.1	0	0	1.9	0	0	0	0	0
ST	0	0	0	0	0	0	0	0	0	0	0	0	0	0	6.1
GM	19.4	23.9	0	0	0	0	0	0	0	0	0	0	0	0	0
AMK	0	0	0	0	1.0	0	3.3	0	0	0	0	0	1.6	0	0
CIP	0	0	0	0	2.0	0	1.1	0	0	0	0	0	0.0	0	0
TC	27.8	32.6	0	0	3	0	0	0	1.2	15.4	0	0	1.6	0	6.1
TGC	0	0	0	0	0	0	0	0	0	0	0	0	0	0	0
FOM	0	0	0	0	0	0	0	0	0	0	0	0	0	0	0
CP	8.3	23.9	0	0	0	0	0	0	0	0	0	0	0	0	0

**Table 3:** Patterns of antimicrobial resistance phenotypes for *E. coli* strains isolated from BC and DCs in the study, with pattern codes.

	Antibiogram Patterns	Number of Isolates	Percentage of Resistance (%)
BC	TC	6	3.9
(n = 152)	CAZ	1	0.7
	ABP + GM + TC	18	11.8
	ABP + TC + CP	2	1.3
	ABP + GM + TC + CP	14	9.2
DCs	TC	13	1.5
(n = 884)	CIP	4	0.5
	AMK	4	0.5
	ABP	1	0.1
	T/P	1	0.1
	ABP + ST + TC	4	0.5
	ABP + T/P + AMK	1	0.1

**Table 4:** Detection of antibiotic resistance genes by head.

Name	BC				DCs			
	B1 (n = 6)	B3 (n = 6)	B5 (n = 7)	Total (n = 19)	D1 (n = 0)	D3 (n = 2)	D8 (n = 5)	Total (n = 7)
*bla*_CTX-M-1_	0	0	0	0	0	0	0	0
*bla*_CTX-M-2_	0	0	0	0	0	0	0	0
*bla*_CTX-M-8_	0	0	0	0	0	0	0	0
*bla*_CTX-M-9_	0	0	0	0	0	0	0	0
*bla*_TEM_	5 (83.3%)	6 (100%)	5 (71.4%)	16 (84.2%)	0	0	3 (60%)	3(42.8)
	n = 10	n = 8	n = 7	n = 25	n = 12	n = 0	n = 5	n = 17
*tet*A	0	0	0	0	8 (66.7%)	0	4 (80%)	12 (70.6%)
*tet*B	5 (50%)	2 (25%)		7 (28%)	0	0	0	0
*tet*C	0	0	0	0	0	0	0	0
*tet*D	0	0	0	0	0	0	0	0
*tet*E	0	0	0	0	0	0	0	0
*tet*G	0	0	0	0	0	0	0	0
*tet*J	0	0	0	0	0	0	0	0
*tet*M	0	0	0	0	0	0	0	0
*tet*W	0	0	0	0	0	0	0	0

**Table 5:** Detection of antibiotic resistance genes monthly.

Name	BC				DCs										
	Oct-22	Nov-22	Dec-22	Jan-23	Oct-22	Nov-22	Dec-22	Jan-23	Apr-23	May-23	Jun-23	Jul-23	Aug-23	Sep-23	Nov-23
	n = 8	n = 11	n = 0	n = 0	n = 1	n = 1	n = 1	n = 0	n = 0	n = 0	n = 0	n = 0	n = 0	n = 0	n = 4
*bla*_CTX-M-1_	0	0	0	0	0	0	0	0	0	0	0	0	0	0	0
*bla*_CTX-M-2_	0	0	0	0	0	0	0	0	0	0	0	0	0	0	0
*bla*_CTX-M-8_	0	0	0	0	0	0	0	0	0	0	0	0	0	0	0
*bla*_CTX-M-9_	0	0	0	0	0	0	0	0	0	0	0	0	0	0	0
*bla*_TEM_	7 (87.5%)	9 (81.8%)	0	0	0	0	0	0	0	0	0	0	0	0	3 (75%)
	n = 10	n = 15	n = 0	n = 0	n = 3	n = 0	n = 0	n = 0	n = 1	n = 8	n = 0	n = 0	n = 1	n = 0	n = 4
*tet*A	0	0	0	0	0	0	0	0	0	8 (100%)	0	0	0	0	3 (75%)
*tet*B	3 (30%)	4 (26.7%)	0	0	0	0	0	0	0	0	0	0	0	0	0
*tet*C	0	0	0	0	0	0	0	0	0	0	0	0	0	0	0
*tet*D	0	0	0	0	0	0	0	0	0	0	0	0	0	0	0
*tet*E	0	0	0	0	0	0	0	0	0	0	0	0	0	0	0
*tet*G	0	0	0	0	0	0	0	0	0	0	0	0	0	0	0
*tet*J	0	0	0	0	0	0	0	0	0	0	0	0	0	0	0
*tet*M	0	0	0	0	0	0	0	0	0	0	0	0	0	0	0
*tet*W	0	0	0	0	0	0	0	0	0	0	0	0	0	0	0

## Data Availability

The original data presented in the study are included in the article. If you need any further information, you can contact the corresponding author.

## Appendix A

**Table A1:** Primers and amplification programs for the detection of *bla*_CTX-M-1, 2, 8, 9_, *bla*_TEM,_ and *tet*A, B, C, D, E, G, J, M, and W.

Gene	Primer	Sequence	Product Size (bp)	Reference	Amplification Program
*uidA*	F	TGGTAATTACCGACGAAAACGGC	162	[10]	Initial denaturation: 95 °C, 3 min Denaturation: 95 °C, 30 s Annealing: 58 °C, 30 s Elongation: 72 °C, 1 min 35 cycles Final extension: 74 °C, 10 min
	R	ACGCGTGGTTACAGTCTTGCG			
*bla*_CTX-M-1_	F	TTAGGAARTGTGCCGCTGYA	688	[13]	Initial denaturation: 94 °C, 10 min Denaturation: 94 °C, 40 s Annealing: 60 °C, 40 s Elongation: 72 °C, 1 min 30 cycles Final extension: 72 °C, 1 min
	R	CGATATCGTTGGTGGTRCCAT			
*bla*_CTX-M-2_	F	CGTTAACGGCACGATGAC	404		
	R	CGATATCGTTGGTGGTRCCAT			
*bla*_CTX-M-8_	F	AACRCRCAGACGCTCTAC	326		
	R	TCGAGCCGGAASGTGTYAT			
*bla*_CTX-M-9_	F	TCAAGCCTGCCGATCTGGT	561		
	R	TGATTCTCGCCGCTGAAG			
*bla*_TEM_	F	CATTTCCGTGTCGCCCTTATTC	800		
	R	CGTTCATCCATAGTTGCCTGAC			
*tet*A	F	TTGGCATTCTGCATTCACTC	494	[14]	Initial denaturation: 95 °C, 2 min Denaturation: 96 °C, 30 s Annealing: 60 °C, 30 s Elongation: 72 °C, 30 s 30 cycles Final extension: 72 °C, 10 min
	R	GTATAGCTTGCCGGAAGTCG			
*tet*B	F	CAGTGCTGTTGTTGTCATTAA	571		
	R	GCTTGGAATACTGAGTGTAA			
*tet*D	F	GCAAACCATTACGGCATTCT	546		
	R	GATAAGCTGCGCGGTAAAAA			
*tet*E	F	TATTAACGGGCTGGCATTTC	544		
	R	AGCTGTCAGGTGGGTCAAAC			
*tet*M	F	ACACGCCAGGACATATGGAT	536		
	R	ATTTCCGCAAAGTTCAGACG			
*tet*W	F	GGGAAATTGTTCGGACAGAC	549		
	R	AACGGATACCATCCCTGACA			
*tet*C	F	GCGGGATATCGTCCATTCCG	207	[15]	Initial denaturation: 94 °C, 5 min Denaturation: 94 °C, 5 s Annealing: 68 °C, 10 s 25 cycles Final extension: 68 °C, 7 min
	R	GCGTAGAGGATCCACAGGACG			
*tet*G	F	GCAGAGCAGGTCGCTGG	134		
	R	CCYGCAAGAGAAGCCAGAAG			
*tet*J	F	CGAAAACAGACTCGCCAATC	184		
	R	TCCATAATGAGGTGGGGC			

## References

[1] World Health Organization (WHO) (2023). Antimicrobial Resistance. https://www.who.int/news-room/fact-sheets/detail/antimicrobial-resistance.

[2] O’Neill J.I.M. (2014). Antimicrobial resistance: Tackling a crisis for the health and wealth of nations. Rev. Antimicrob. Resist..

[3] Bishop M. (2017). Global disruption of antibiotic-resistant bacteria. Public health post.

[4] Mulchandani R., Wang Y., Gilbert M., Van Boeckel T.P. (2023). Global trends in antimicrobial use in food-producing animals: 2020 to 2030. PLOS Glob. Public Health.

[5] (2022). Nippon AMR one health report (NAOR 2022) JVARM. https://amr-onehealth.ncgm.go.jp/en/.

[6] Asai T. (2008). Antimicrobial resistance monitoring program in food-producing animals in Japan. J. Vet. Epidemiol..

[7] Fujimoto K., Kawasaki M., Abe R., Yokoyama T., Haga T., Sugiura K. (2021). Establishing defined daily doses (DDDs) for antimicrobial agents used in pigs, cattle, and poultry in Japan and comparing them with European DDD values. PLoS ONE.

[8] Sawant A.A., Hegde N.V., Straley B.A., Donaldson S.C., Love B.C., Knabel S.J., Jayarao B.M. (2007). Antimicrobial-resistant enteric bacteria from dairy cattle. Appl. Environ. Microbiol..

[9] Centers for Disease Control and Prevention (CDC) (2019). Antibiotic Resistance Threats in the United States, 2019.

[10] Suzuki Y., Hiroki H., Xie H., Nishiyama M., Sakamoto S.H., Uemura R., Nukazawa K., Ogura Y., Watanabe T., Kobayashi I. (2022). Antibiotic-resistant *Escherichia coli* isolated from dairy cows and their surrounding environment on a livestock farm practicing prudent antimicrobial use. Int. J. Hyg. Environ. Health.

[11] Bej A.K., Steffan R.J., DiCesare J., Haff L., Atlas R.M. (1990). Detection of coliform bacteria in water by polymerase chain reaction and gene probes. Appl. Environ. Microbiol..

[12] Clinical Laboratory Standard Institute Performance Standards for Antimicrobial Susceptibility Testing. 2023, Volume 2023. http://em100.edaptivedocs.net/.

[13] Dallenne C., Da Costa A.D., Decré D., Favier C., Arlet G. (2010). Development a set of multiplex PCR assays for the detection of genes encoding important beta-lactamases in Enterobacteriaceae. J. Antimicrob. Chemother..

[14] Call D.R., Bakko M.K., Krug M.J., Roberts M.C. (2003). Identifying antimicrobial resistance genes with DNA microarrays. Antimicrob. Agents Chemother..

[15] Aminov R.I., Chee-Sanford J.C., Garrigues N., Teferedegne B., Krapac I.J., White B.A., Mackie R.I. (2002). Development, validation, and application of PCR primers for detection of tetracycline efflux genes of Gram-negative bacteria. Appl. Environ. Microbiol..

[16] Bogaard A.E., Stobberingh E.E. (2000). Epidemiology of resistance to antibiotics. Links between animals and humans. Int. J. Antimicrob. Agents.

[17] Sayah R.S., Kaneene J.B., Johnson Y., Miller R. (2005). Patterns of antimicrobial resistance observed in *Escherichia coli* isolates obtaind from domestic- and wild-animal fecal samples, human septage, and surface water. Appl. Environ. Microbiol..

[18] Sobur M.A., Sabuj A.A.M., Sarker R., Rahman A.M.M.T., Kabir S.M.L., Rahman M.T. (2019). Antibiotic-resistant *Escherichia coli* and Salmonella spp. associated with dairy cattle and farm environment having public health significance. Vet. World.

[19] Cheney T.E., Smith R.P., Hutchinson J.P., Brunton L.A., Pritchard G., Teale C.J. (2015). Cross-sectional survey of antibiotic resistance in *Escherichia coli* isolated from diseased farm livestock in England and Wales. Epidemiol. Infect..

[20] Hennessey M., Whatford L., Payne-Gifford S., Johnson K.F., Van Winden S., Barling D., Häsler B. (2020). Antimicrobial & antiparasitic use and resistance in British sheep and cattle: A systematic review. Prev. Vet. Med..

[21] Morris C., Wickramasingha D., Abdelfattah E.M., Pereira R.V., Okello E., Maier G. (2023). Prevalence of antimicrobial resistance in fecal *Escherichia coli* and *Enterococcus* spp. isolates from beef cow-calf operations in northern California and associations with farm practices. Front. Microbiol..

[22] Surette M.D., Wright G.D. (2017). Lessons from the environmental antibiotic resistome. Annu. Rev. Microbiol..

[23] Schmid A., Hörmansdorfer S., Messelhäusser U., Käsbohrer A., Sauter-Louis C., Mansfeld R. (2013). Prevalence of Extended-spectrum-βlactamase-producing *Escherichia coli* on Bavarian dairy and beef cattle farms. Appl. Environ. Microbiol..

[24] Wilson J.B., McEwen S.A., Clarke R.C., Leslie K.E., Wilson R.A., Waltner-Toews D., Gyles C.L. (1992). Distribution and characteristics of verocytotoxigenic *Escherichia coli* isolated from Ontario dairy cattle. Epidemiol. Infect..

[25] Call D.R., Davis M.A., Sawant A.A. (2008). Antimicrobial resistance in beef and dairy cattle production. Anim. Health Res. Rev..

[26] (2023). National Action Plan on Antimicrobial Resistance (AMR), 2023–2027. https://www.mhlw.go.jp/content/10900000/001096228.pdf.

[27] Yamamoto S., Iwabuchi E., Hasegawa M., Esaki H., Muramatsu M., Hirayama N., Hirai K. (2013). Prevalence and molecular epidemiological characterization of antimicrobial-resistant *Escherichia coli* isolates from Japanese black beef cattle. J. Food Prot..

[28] Wagner B.A., Dargatz D.A., Salman M.D., Morley P.S., Wittum T.E., Keefe T.J. (2002). Comparison of sampling techniques for measuring the antimicrobial susceptibility of enteric *Escherichia coli* recovered from feedlot cattle. Am. J. Vet. Res..

[29] Carson C.A., Reid-Smith R., Irwin R.J., Martin W.S., McEwen S.A. (2008). Antimicrobial resistance in generic fecal *Escherichia coli* from 29 beef farms in Ontario. Can. J. Vet. Res..

[30] Lim S.K., Lee H.S., Nam H.M., Cho Y.S., Kim J.M., Song S.W., Park Y.H., Jung S.C. (2007). Antimicrobial resistance observed in *Escherichia coli* strains isolated from fecal samples of cattle and pigs in Korea during 2003–2004. Int. J. Food Microbiol..

[31] Gow S.P., Waldner C.L., Rajić A., McFall M.E., Reid-Smith R. (2008). Prevalence of antimicrobial resistance in fecal generic *Escherichia coli* isolated in western Canadian beef herds. Part II. Cows and cow-calf pairs. Can. J. Vet. Res..

[32] Khachatryan A.R., Hancock D.D., Besser T.E., Call D.R. (2004). Role of calf-adapted *Escherichia coli* in maintenance of antimicrobial drug resistance in dairy calves. Appl. Environ. Microbiol..

[33] Poirel L., Madec J.Y., Lupo A., Schink A.K., Kieffer N., Nordmann P., Schwarz S. (2018). Antimicrobial resistance in *Escherichia coli*. Microbiol. Spec..

[34] Braun S.D., Ahmed M.F.E., El-Adawy H., Hotzel H., Engelmann I., Weiß D., Monecke S., Ehricht R. (2016). Surveillance of extended-spectrum beta-lactamase producing *Escherichia coli* in dairy cattle farms in the Nile Delta, Egypt. Front. Microbiol..

[35] Ohnishi M., Sawada T., Harada K., Esaki H., Shimura K., Marumo K., Takahashi T. (2012). Occurrence of bovine mastitis caused by CTX-M-2 β-lactamase producing Klebsiella pneumoniae. J. Vet. Epidemol.

[36] Yue S., Zhang Z., Liu Y., Zhou Y., Wu C., Huang W., Chen N., Zhu Z. (2021). Phenotypic and molecular characterizations of multidrug-resistant diarrheagenic, *E. coli* of calf origin. Anim. Dis..

